# Socioeconomic, Temporal and Regional Variation in Body Mass Index among 188,537 Swiss Male Conscripts Born between 1986 and 1992

**DOI:** 10.1371/journal.pone.0096721

**Published:** 2014-05-12

**Authors:** Radoslaw Panczak, Marcel Zwahlen, Ulrich Woitek, Frank J. Rühli, Kaspar Staub

**Affiliations:** 1 Centre for Evolutionary Medicine, Institute of Anatomy, University of Zurich, Zurich, Switzerland; 2 Institute of Social and Preventive Medicine, University of Bern, Bern, Switzerland; 3 Department of Economics, University of Zurich, Zurich, Switzerland; Cuny, United States of America

## Abstract

**Background:**

Rising levels of overweight and obesity are important public-health concerns worldwide. The purpose of this study is to elucidate their prevalence and trends in Switzerland by analyzing variations in Body Mass Index (BMI) of Swiss conscripts.

**Methods:**

The conscription records were provided by the Swiss Army. This study focussed on conscripts 18.5–20.5 years of age from the seven one-year birth cohorts spanning the period 1986–1992. BMI across professional status, area-based socioeconomic position (abSEP), urbanicity and regions was analyzed. Two piecewise quantile regression models with linear splines for three birth-cohort groups were used to examine the association of median BMI with explanatory variables and to determine the extent to which BMI has varied over time.

**Results:**

The study population consisted of 188,537 individuals. Median BMI was 22.51 kg/m^2^ (22.45–22.57 95% confidence interval (CI)). BMI was lower among conscripts of high professional status (−0.46 kg/m^2^; 95% CI: −0.50, −0.42, compared with low), living in areas of high abSEP (−0.11 kg/m^2^; 95% CI: −0.16, −0.07 compared to medium) and from urban communities (−0.07 kg/m^2^; 95% CI: −0.11, −0.03, compared with peri-urban). Comparing with Midland, median BMI was highest in the North-West (0.25 kg/m^2^; 95% CI: 0.19–0.30) and Central regions (0.11 kg/m^2^; 95% CI: 0.05–0.16) and lowest in the East (−0.19 kg/m^2^; 95% CI: −0.24, −0.14) and Lake Geneva regions (−0.15 kg/m^2^; 95% CI: −0.20, −0.09). Trajectories of regional BMI growth varied across birth cohorts, with median BMI remaining high in the Central and North-West regions, whereas stabilization and in some cases a decline were observed elsewhere.

**Conclusions:**

BMI of Swiss conscripts is associated with individual and abSEP and urbanicity. Results show regional variation in the levels and temporal trajectories of BMI growth and signal their possible slowdown among recent birth cohorts.

## Introduction

After three decades of steady increase, overweight and obesity (OWOB) prevalence has reached the level of a global pandemic [1]: 65% of the world's population live in countries where OWOB kill more people than does underweight [2]. In 2008, 1.5 billion adults were overweight. It has been projected that these numbers will continue to increase until the year 2030 [3]. Excess body weight is a major health concern, contributing as it does to an increase in the risk of morbidity and mortality. Overweight and, particularly, obesity are associated with many chronic, non-communicable diseases (e.g., diabetes type 2, hypertension and other cardiovascular diseases, various cancers etc.), even among young people [4], [5].

There has been considerable research done in Switzerland showing that OWOB prevalence has increased significantly since the early 1990s [6]–[9]. Several studies have reported socioeconomic (as measured by occupational status, income or education) and regional differences in the prevalence of OWOB in Switzerland [7], [8], [10]–[14]. Recently it was estimated that approximately 27,000 cases of type 2 diabetes, 63,000 cases of high blood pressure and 37,000 cases of dyslipidaemia could have been avoided if OWOB in Switzerland had remained at their 1992 levels [4]. OWOB, their co-morbidities and their health consequences represented 11% of total Swiss healthcare expenses in 2006, thus creating a considerable economic and public-health burden [15]. Despite the fact that OWOB are acknowledged to be a serious problem, there is a lack of nationally measured, longitudinal samples and thus of objective, precise and representative information on their prevalence in Switzerland [6], [16], [17]. The majority of findings are based on sporadic, irregular surveys that are based, in turn, on regionally, demographically or socioeconomically restricted samples. These studies are limited in their representativeness (due to sample selection bias, sample size and a decline in the number of participants), and tend to over- or underestimate the actual prevalence of OWOB [7].

Depending on which sex, age, ethnic or socioeconomic group was surveyed and which body-shape measurement methods were used, the actual overweight prevalence varied between 25% and 50%. In particular, there is considerable inter-study variability of obesity prevalence among the Swiss Health Survey (SHS) data, various epidemiological studies and sporadic regional surveys [6], [10], [11], [16]–[22]. As is the case with the majority of epidemiological investigations, Swiss studies estimated the prevalence of OWOB by calculating and categorizing Body Mass Index (BMI  =  kg/m^2^) derived from height and weight [23]. In most Swiss surveys, such as the SHS and the Swiss Household Panel (SHP), these calculations rely on self-reported weight and height, which can lead to a misclassification bias and distort the relationship between obesity and disease or death because individuals tend to overestimate their height and underestimate their weight [24]–[26]. Despite heterogeneous populations and methodologies, the results from studies based on SHS and schoolchildren-monitoring data indicate that the OWOB prevalence among both adults and children over the past five years has begun to level off, suggesting that increased awareness combined with mainly school-based programmes aimed at physical activity and healthful eating habits are beginning to pay off [10], [27]–[31]. However, the regional and temporal dynamics of these processes remain largely unknown.

Finland, Norway, Denmark, Austria and Switzerland are the only countries of Western Europe that still rely on full, regular conscription of their citizens in their military structure. The conscription process includes standardized anthropometric measurements that take place during a medical examination that is mandatory for all young men, including those subsequently declared to be unfit for military service. These data yield a yearly picture of the anthropometric status of young men at a prescribed age [32]. Although the conscription process was not designed with epidemiological studies in mind, its data have been successfully used for medical and epidemiological research in Switzerland [33], [34], Austria [35] and Germany [36]. Despite its focus on male populations, the OWOB status of conscripts is a valuable tool for public-health research for two reasons: because being overweight in adolescence increases the risk of being overweight as an adult, and because particularly men's morbidity and mortality risks increase with age [37], [38]. The main aim of the current study is to investigate the socioeconomic, temporal and regional differences in objectively measured BMI among the most recent birth cohorts of Swiss conscripts.

## Materials and Methods

### Swiss conscription

The mandatory, multi-day recruitment concept of the Swiss Army (a draft army composed of male Swiss citizens), a concept instituted in 1875, was renewed and expanded in 2004. The regulations specify that all young men are called to conscription in the year they turn 19. However, both earlier and later conscription are possible upon request. In addition, approximately 200 women voluntarily join the Army each year. The medical assessments that are part of the conscription process include the measurement of anthropometric data (height and weight, rounded to integers) and the recording of socioeconomic status (indicated by current profession) and place of residence of every conscript, including those who subsequently receive either a deferral or an exemption. These assessments are made under professional medical supervision at six dedicated conscription centres (Lausanne, Sumiswald, Windisch, Rüti, Mels, Monte Ceneri), with identical qualitative standards for technical equipment and organizational structures (Bundesgesetz über die Armee und die Militärverwaltung, Militärgesetz MG, 510.10, Art. 2; MG Art. 9, Verordnung über die Rekrutierung VREK, 511.11, Art. 3 and Art. 9). All measurements are immediately recorded in the medical-information system of the Swiss Army (Medizinisches Informationssystem der Armee, MEDISA), and can then be accessed by Army personnel. These records cover more than 90% of the annual male birth cohorts, making possible the investigation of socioeconomic, temporal and regional differences across the country [33], [34]. According to official statements by the Swiss Army [39], the medical causes of those young men classified as unfit for service in absentia (up to 10%) include the full range of severe diseases and of severe physical and psychological disabilities. Because of the size of this group and the fact that the list of all possible reasons is not limited to diseases linked to body shape, absenteeism was not considered to affect the BMI distribution in a considerable way.

### Data

Fully anonymous, individual conscription records for the period of 1 January 2004 to 31 December 2012 were provided by the Swiss Army (Logistikbasis der Armee, LBA San) under contractual agreement with the study authors. The received data included date of birth, date of conscription, height, weight, current occupation (recorded as free-text entry), postcode of place of residence and stage of conscription (first, regular visit versus reassessment).

### Data-availability statement

The data and the permission to use them are available from the Swiss Armed Forces (Logistikbasis der Armee - LBA San [39]) upon submission and approval of a study protocol.

### Ethics statement

According to the signed bilateral data contract, the Swiss Armed Forces fully anonymized the records by removing all names, social security numbers and exact residential addresses prior to the data delivery to the study authors. Swiss conscription is mandatory and the anthropometric measurements used in this study are non-clinical, governmental data; therefore no informed consent is required [40]. According to Swiss federal law (Bundesgesetz über die militärischen Informationssysteme MIG, BG 510.91, Art. 2, 9, 24–29), the Swiss Army is authorized to make the data accessible for academic research in anonymous form. When dealing with anonymized and non-clinical data, no additional ethical approval is needed for analyses based on such governmental data (Swiss data privacy act, SR 235.1; 19.6.1992).

### Study population

We included male conscripts appearing for the first, regular assessment in the recruitment centre. We excluded those whose date of birth or postcode of place of residence was missing or implausible and also those with height and weight values beyond a certain range (130 cm > height >220 cm; 30> weight >200 kg; apart from one conscript whose weight was recorded as 500 kg – most probably a typing error – none of the conscripts was found in the 200+kg range). Additionally, the dataset was checked for implausible BMI values but none were found.

We included individuals born between 1986 and 1992 and conscripted when 18.5–20.5 years old. These criteria guaranteed that the age structure of the study population was homogeneous and minimized the risk of including those conscripts who had requested permission to undergo conscription either before or after the year in which they turned 18. Additionally, in regard to age range every birth cohort is fully represented in the dataset. As a sensitivity analysis we repeated the modelling after having modified it in two respects, extending the dataset to conscripts aged 18.0–21.0, and limiting it to 19.0–20.0.

### Representativeness

Currently there is no dataset available that can be used to assess with precision the degree to which the conscript population is representative of the total population of young men in Switzerland. The conscript counts can be compared with the total-live-birth counts or with an estimate of the total number of individuals of a certain age living in a given calendar year. However, on account of internal and external migration and deaths that occurred between the time of birth and the time of conscription, both of these methods will lead to an inaccurate estimation of the representativeness of the conscript population. In order to assess the representativeness of our dataset, we compared the counts of male conscripts in selected birth cohorts with population estimates provided by the Population and Households Statistics [41]. We used data on the total number of live births for the years matching the conscript population and estimates of the mid-year count of 17-year-old males for a given calendar year.

### Spatial linkage

Swiss postcodes change over time and do not correspond exactly to administrative boundaries. The postcode of the place of residence of each conscript was standardized to the state on 1 January 2013 and assigned the SFSO code of community (*Gemeinde*, the lowest level of administrative subdivision) inside which it lay, in order to provide a link between postal and administrative geographies. In the case of postcodes the boundaries of which overlapped with two or more communities, the community with the largest populations overlap was used.

### Variables

We calculated Body Mass Index (BMI  =  weight [kg]/height [m]^2^) and used the World Health Organization's categories for the definition of OWOB [42]. We calculated age at conscription on the basis of date of birth and date of conscription. The birth cohort was determined on the basis of year of birth. We converted free-text entry of the current occupation to the International Standard Classification of Occupations (ISCO-08) code as described by the International Labour Organization [43]. The ISCO major groups were then collapsed to form three hierarchical categories, ‘Low’ (ISCO major groups 7 to 9), ‘Medium’ (ISCO major groups 3 to 6) and ‘High’ (ISCO major groups 1 and 2, and students) professional status. We created separate categories for individuals who were still in school (‘Pupils’) and for those cases in which data on occupational status were insufficient or missing (‘Imprecise’).

We calculated median value of the Swiss neighbourhood index of socioeconomic position (Swiss-SEP; [44]) for each of the postcodes and assigned it to the individual records on the basis of postcode of place of residence. Median postcode Swiss-SEP was then categorized into tertiles, 1, 2. and 3, in order of increasing socioeconomic status.

**Figure 1:**
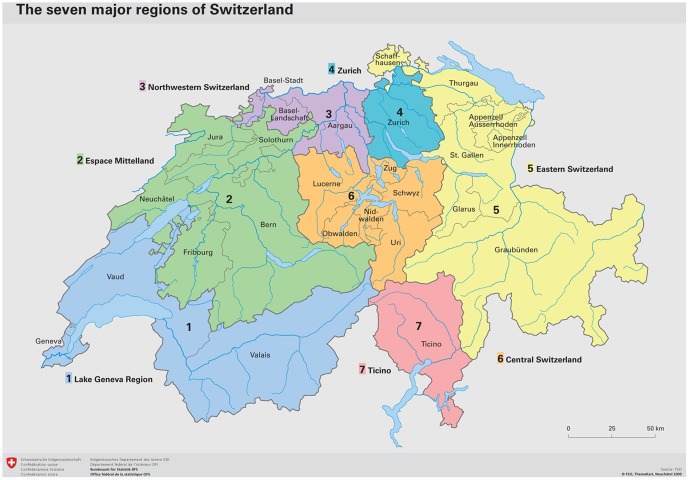
The seven major regions *(Grossregione*n) of Switzerland (Source: Swiss Federal Statistical Office SFSO, ThemaKart).

Each conscript was assigned to one of seven regions (*Grossregion*) on the basis of community of residence: ‘Midland,’ ‘North-West,’ ‘East,’ ‘Lake Geneva,’ ‘Ticino,’ ‘Central’ and ‘Zurich’ (**Figure 1**). These regional divisions are compatible with the second level of the European Union's ‘Nomenclature of territorial units for statistics’ (NUTS 2), a hierarchical, regional classification system that divides Europe into basic regions for the application of regional policies. We defined the urbanicity level in accordance with the classification of the Swiss Federal Statistical Office [45].

### Statistical analyses

To describe the distribution of BMI across birth cohorts and independent variables, we used frequencies (for BMI categories) and means, standard deviations (SD), medians and inter-quartile-ranges (IQR) (for BMI). To assess associations of regional and socioeconomic variables at the 50^th^ percentile (median) of BMI, we used quantile regression [46], with BMI as the outcome. The quantile regression has been successfully used in previous studies of BMI. One of its advantages is that it minimizes the impact of the outliers and skewness of the data on the estimated coefficients [47]. In order to adjust models for time and assess the interaction between time and independent variables, we used piecewise models, splitting the birth years of conscripts into three groups, with linear splines for the periods 1986–87, 1988–89 and 1990–92. We applied the Wald test for interaction among the independent variables and these three birth-year periods, assessing the composite linear hypothesis that all of the interaction parameters are jointly zero. Stata version 13 (Stata Corporation, College Station, TX, USA) was used for all of the statistical analyses.

## Results

### Study population

The initial sample comprised 325,747 records. Of these overall data, we considered individuals fulfilling all of the following criteria at the same time: Regular (N = 313,666; 96.3% of the initial sample) male (N = 323,759; 99.4%) conscripts with complete information on date of birth (N = 325,719; >99.9%) and postcode of place of residence (N = 325,739; >99.9%), with plausible height and weight values (N = 325,674; >99.9%), between 18.5 and 20.5 years of age at the time of conscription (N = 232,707; 71.4%), as well as having been born between 1986 and 1992 (N = 265,731; 81.6%).

The analyzed sample consisted of 188,537 individuals fulfilling all mentioned criteria at the same time, with each birth cohort contributing between 25,512 (13.5%) and 28,199 (15.0%) conscripts (**Table 1**). The professional status, as defined by current occupation, of more than a third of the conscripts (N = 72,761, 38.6%) was low; as for neighbourhood SEP, the residences of nearly half of them (N = 87,366, 46.3%) were located in the middle tertile index. A third of the study population (N = 61,480, 32.6%) came from rural areas. The regions of Midland and East Switzerland contributed the largest number of conscripts (49,498, 26.3% and 30,727, 16.3%, respectively), followed by the Lake Geneva, Zurich, North-West and Ticino regions. The distribution of the conscripts across regions and urbanicity levels resembled that of the Swiss population in general.

**Table 1:** Distribution of Body Mass Index (BMI) (mean, standard deviation (SD), median and inter-quartile range (IQR)) and frequencies of major BMI categories across year of birth and contextual variables of Swiss conscripts.

Variable	BMI category								Total					
	*<18.5*		*18.5*–*24.99*		*25.0*–*29.9*		*> = 30*							
	*No.*	*Row %*	*No.*	*Row %*	*No.*	*Row %*	*No.*	*Row %*	*No.*	*Column %*	*Mean*	*SD*	*Median*	*IQR*
***Year of birth***														
1986	1′226	4.8%	19′033	74.6%	4′146	16.3%	1′107	4.3%	25′512	13.5%	22.98	3.56	22.34	20.65–24.54
1987	1′185	4.5%	19′776	74.4%	4′342	16.3%	1′283	4.8%	26′586	14.1%	23.10	3.65	22.41	20.75–24.62
1988	1′117	4.1%	20′171	73.6%	4′727	17.3%	1′386	5.1%	27′401	14.5%	23.21	3.68	22.53	20.81–24.76
1989	957	3.6%	19′379	72.6%	4′872	18.3%	1′480	5.5%	26′688	14.2%	23.35	3.74	22.64	20.90–24.91
1990	1′006	3.7%	19′600	71.7%	5′169	18.9%	1′576	5.8%	27′351	14.5%	23.43	3.75	22.72	20.97–25.03
1991	1′029	3.6%	20′045	71.1%	5′489	19.5%	1′636	5.8%	28′199	15.0%	23.45	3.80	22.74	20.94–25.06
1992	954	3.6%	19′185	71.6%	5′112	19.1%	1′549	5.8%	26′800	14.2%	23.36	3.73	22.68	20.90–24.93
***Professional status***														**-**
High	1′991	4.7%	32′047	76.1%	6′454	15.3%	1′613	3.8%	42′105	22.3%	22.87	3.44	22.31	20.66–24.36
Medium	1′738	4.0%	31′206	71.8%	8′132	18.7%	2′371	5.5%	43′447	23.0%	23.34	3.74	22.68	20.90–24.96
Low	2′280	3.1%	51′558	70.9%	14′434	19.8%	4′489	6.2%	72′761	38.6%	23.55	3.78	22.84	21.05–25.20
Pupil	840	4.8%	13′295	76.3%	2′582	14.8%	697	4.0%	17′414	9.2%	22.88	3.54	22.28	20.60–24.34
Imprecise	625	4.9%	9′083	70.9%	2′255	17.6%	847	6.6%	12′810	6.8%	23.38	4.13	22.53	20.68–25.03
***Swiss-SEP***														
1. tertile	1′870	3.8%	34′886	71.2%	9′281	18.9%	2′946	6.0%	48′983	26.0%	23.42	3.84	22.66	20.90–25.03
2. tertile	3′382	3.9%	63′543	72.7%	15′669	17.9%	4′772	5.5%	87′366	46.3%	23.29	3.73	22.59	20.83–24.86
3. tertile	2′222	4.3%	38′760	74.3%	8′907	17.1%	2′299	4.4%	52′188	27.7%	23.12	3.54	22.52	20.80–24.67
***Urbanicity***														
Rural	2′124	3.5%	44′193	71.9%	11′555	18.8%	3′608	5.9%	61′480	32.6%	23.40	3.76	22.66	20.94–24.96
Peri-urban	3′250	4.0%	59′185	72.9%	14′577	17.9%	4′216	5.2%	81′228	43.1%	23.26	3.69	22.59	20.83–24.84
Urban	2′100	4.6%	33′811	73.8%	7′725	16.9%	2′193	4.8%	45′829	24.3%	23.13	3.66	22.47	20.75–24.68
***Region***														
Midland	1′841	3.7%	35′749	72.2%	8′959	18.1%	2′949	6.0%	49′498	26.3%	23.35	3.82	22.59	20.83–24.90
North-West	840	3.3%	18′034	71.7%	4′801	19.1%	1′472	5.9%	25′147	13.3%	23.52	3.78	22.84	21.05–25.09
East	1′246	4.1%	22′657	73.7%	5′320	17.3%	1′504	4.9%	30′727	16.3%	23.09	3.61	22.41	20.75–24.66
Lake Geneva	1′397	5.1%	20′260	73.3%	4′647	16.8%	1′350	4.9%	27′654	14.7%	23.11	3.72	22.41	20.66–24.69
Ticino	334	4.4%	5′601	73.5%	1′323	17.4%	363	4.8%	7′621	4.0%	23.12	3.63	22.5	20.72–24.64
Central	699	3.2%	15′613	72.2%	4′133	19.1%	1′189	5.5%	21′634	11.5%	23.38	3.64	22.72	20.98–24.93
Zurich	1′117	4.3%	19′275	73.4%	4′674	17.8%	1′190	4.5%	26′256	13.9%	23.25	3.60	22.64	20.90–24.82
***Total***	7′474	4.0%	137′189	72.8%	33′857	18.0%	10′017	5.3%	188′537	100.0%	23.27	3.71	22.59	20.83–24.84

### Representativeness

Between 2003 and 2009, the study sample of conscripts 18.5–20.5 years of age represented between 70.0% and 76.1% of the Swiss male population count (at the age of 17) matched by calendar year (**Table S1**). Similarly, the range of representativeness was between 78.3% and 81.6% when comparing the birth years 1986 to 1992 with the total number of live births matched by year of birth. It is noteworthy that the percentages were lower for older cohorts among younger conscripts and for younger cohorts among older conscripts, who were not the part of the analysis.

### Trends of crude BMI and OWOB prevalences across socioeconomic and regional strata

The mean BMI and, in consequence, the prevalence of OWOB increased among the 1986 to 1990 birth cohorts, with the trend levelling off in the two last birth cohorts, those of 1991 and 1992 (**Table 1**). For instance, the mean BMI of the 1986 cohort was 22.98 (SD  = 3.56) as compared with 23.45 (SD  = 3.80) for the 1991 birth cohort and decreased to a mean of 22.36 (SD  = 3.73) for the last analyzed cohort, that of 1992. Similarly, the prevalence of obesity increased from 4,3% in the 1986 birth cohort to 5.8% in the 1990 cohort and remained at this level for both the 1991 and the 1992 ones.

There were marked differences in mean BMI among the socioeconomic and regional subgroups of the study population. The mean BMI was higher among conscripts who were of lower professional status and among those who were living in neighbourhoods of lower SEP and in rural communities (**Table 1**). The differences in mean BMI remained stable across birth cohorts for professional status, Swiss-SEP index and urbanicity. However, the main regions of Switzerland were characterized by differences not only in the level of mean BMI and the prevalence of OWOB (**Table 1**) but also in the temporal trajectories of the growth of mean-BMI values (**Figure S1**, bottom), with the higher values being found in the North-West, Midland and Central regions.

### Association of BMI with socioeconomic and regional characteristics

**Figure 2:**
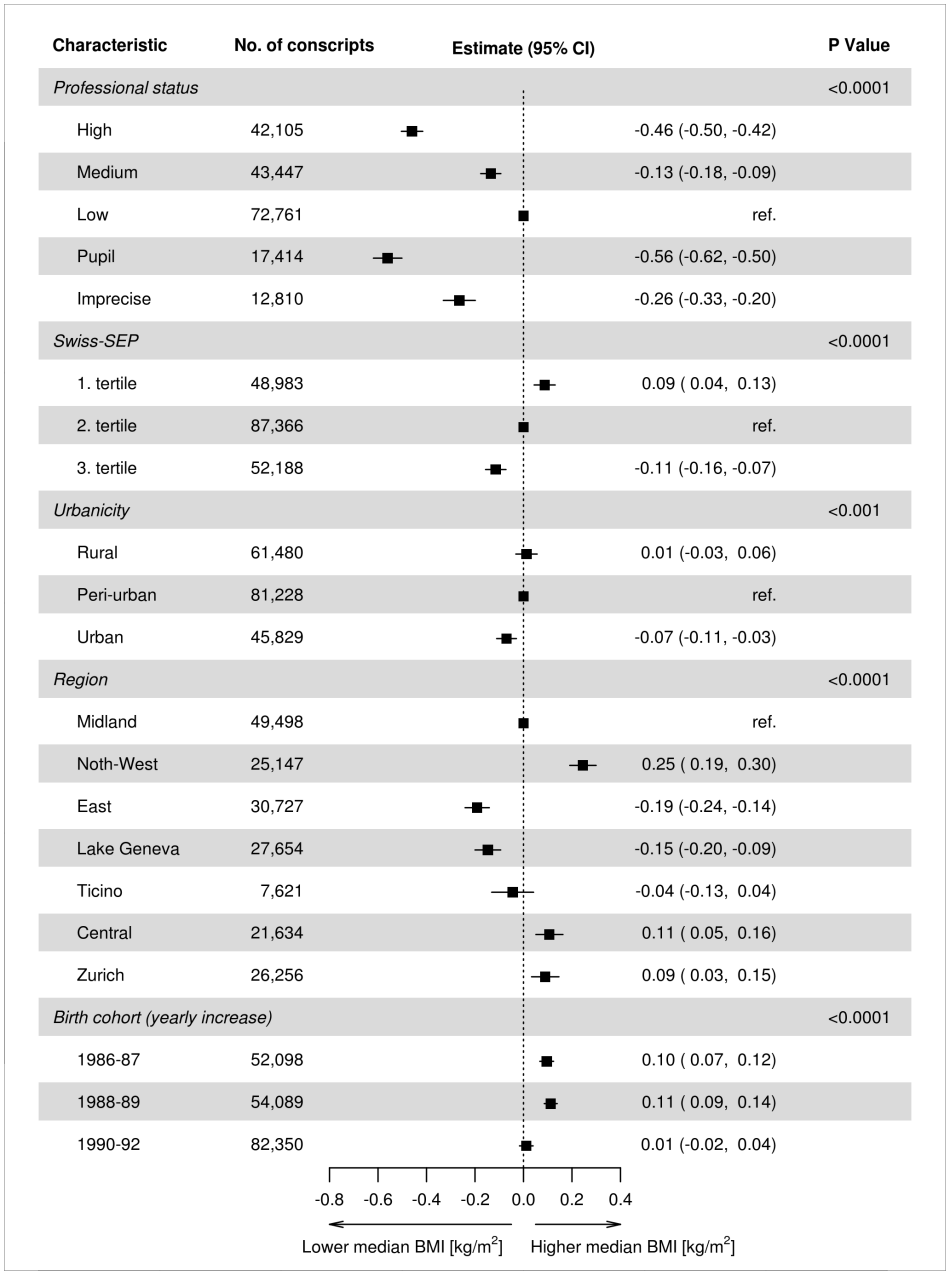
Differences in median BMI (95% confidence intervals (CI)) estimated from the first multivariable quantile regression model of Swiss conscripts across professional status, tertiles of median Swiss-SEP index of postcode of residence, degree of urbanicity and region of residence. Model adjusted for linear splines for birth-year period.

**Figure 2** shows the results of the first piecewise, multivariable quantile regression model of association of median BMI with socioeconomic and regional factors adjusted for year of birth using linear splines. Median BMI was 22.51 kg/m^2^ (22.45–22.57 95% confidence interval (CI)). As in crude estimates of mean BMI (**Table 1**), the median BMI was higher among the lower socioeconomic strata of the conscript population, as measured on both the individual (professional status) and the neighbourhood levels (Swiss-SEP). For instance, the median BMI of the conscripts of ‘High’ professional status was −0.46 kg/m^2^ (−0.50, −0.42 95% CI) lower than that of those in the ‘Low’ group. There was a clear gradient of decrease of median BMI across tertiles of abSEP, with conscripts coming from areas of lowest abSEP having a median BMI −0.11 kg/m^2^ (−0.16, −0.07 95% CI) lower than those of the 2. tertile. The influence of urbanicity was smaller, with conscripts coming from urban areas having a median BMI −0.07 kg/m^2^ (−0.11, −0.03 95% CI) lower than those of rural ones. Median BMI varied sharply among regions: highest in the North-West, Central and Zurich regions and lowest in the East, Lake Geneva and Ticino ones, compared with the Midland region (**Figure 2**). Finally, the yearly increases of BMI among conscripts from the 1986–87 and 1988–89 cohorts (0.10 and 0.11 kg/m^2^, respectively) stabilized among conscripts from the last cohort, born between the years 1990 and 1992 (yearly increase of 0.01 kg/m^2^;−0.02, 0.04 95% CI).

### Trends of BMI across regions and birth cohorts

**Figure 3:**
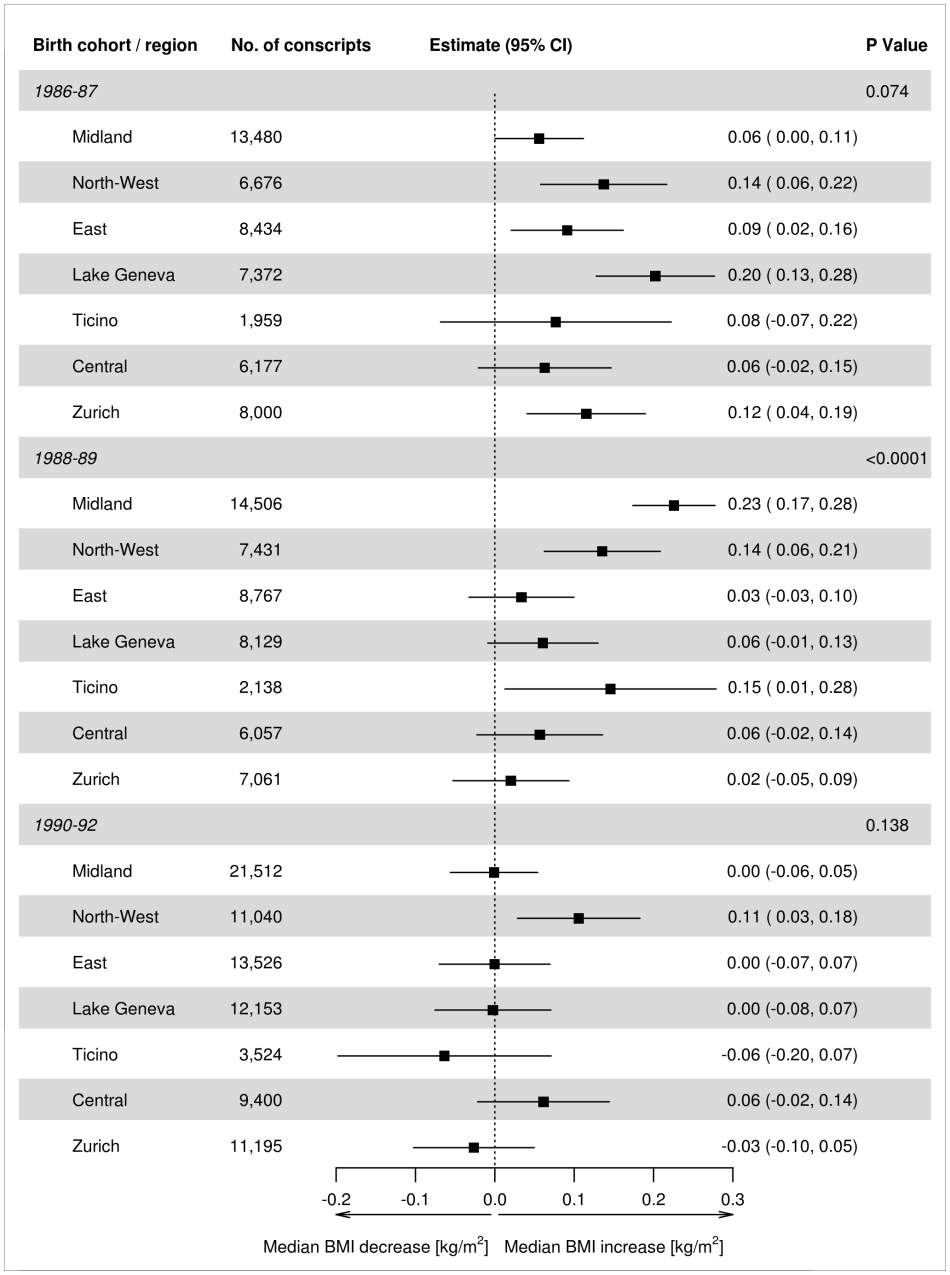
Annual change in median BMI (95% confidence intervals) estimated from the second multivariable quantile regression model of Swiss conscripts across birth cohort and region of residence. Model adjusted for professional status, tertile of median Swiss-SEP index of postcode of residence, degree of urbanicity of community of residence and linear splines for birth-year period and interaction of region with birth-year period.

In regard to the first two birth-cohort groups, we found evidence of interaction between regions and restricted splines (test of interaction *P* = 0.01, P<0.0001 and P = 0.14 for 1986–87, 1988–89 and 1990–92 birth cohorts, respectively), but we found no evidence of interaction between professional status, Swiss-SEP tertile of postcode of residence and the degree of urbanicity of community of residence (all *P* values ≥0.08). **Figure 3** shows results of the second piecewise, multivariable quantile regression model, including interaction term of birth-cohort period and region of residence. The model was adjusted for professional status, Swiss-SEP tertile and the degree of urbanicity. Median BMI was estimated at 22.47 kg/m^2^ (22.39–22.55 95% CI): similar to that of the first model.

Heterogeneity was found in the temporal trajectories of median-BMI change across birth cohorts and regions. The median BMI of the 1986–87 cohorts increased across all regions, the greatest increases being in the Lake Geneva (0.20 kg/m^2^; 0.13–0.28 95% CI) and North-West (0.14 kg/m^2^; 0.06–0.22 95% CI) regions. The median-BMI values of the 1988–89 cohorts decreased in the East, Lake Geneva and Zurich regions, remained stable in the North-West and Central regions and increased in the Midland and Ticino regions. The median BMI of the 1990–92 birth cohorts reversed their upward direction in Ticino and Zurich; however, the estimates failed to reach the conventional levels of statistical significance. Stabilization was observed in three regions: Midland, East and Lake Geneva. Only in the North-West and Central regions did the values remain positive.

## Discussion

The aim of this study was to investigate the temporal, regional and socioeconomic differences in BMI among the most recent birth cohorts of Swiss conscripts. We found divergences in the median levels and temporal trajectories of BMI changes among conscripts from seven major regions of Switzerland and, overall, indications that the increase may be levelling off. As in other studies, the BMI of individuals occupying the lower end of the socioeconomic scale, including those from rural communities, was on average higher than that of their better-off and urban counterparts.

The current study has several strengths. It relied on a large, representative sample and objectively measured data. The size of the sample allowed investigation of the levels and development of the BMI across birth year cohorts as well as the major regions of Switzerland. The findings contribute to the understanding of the OWOB prevalence and of regional and temporal trends among the Swiss population.

It must be acknowledged that the study has five limitations. First, because of the nature of the conscription process, the analyses based on conscription data apply exclusively to male Swiss nationals. However, comparison of these data with those derived from other Swiss sources indicate that the conscript data, featuring the most precise and objective height and weight measurements based on a very large sample, offer information of unparalleled reliability regarding BMI trends among young Swiss males. Second, the fact that information about requests for permission to undergo conscription before or after the prescribed age is not recorded in MEDISA database could, in theory, skew the results; we therefore conducted sensitivity analyses on samples of conscripts 19.0–20.0 and 18.0–21.0 years of age, and found that, in fact, differences in age at conscription had no significant effect on our findings. Third, BMI was the only body-shape measure available in the dataset. BMI is not an ideal measure of body composition since it does not precisely differentiate between weight associated with lean muscle mass and weight associated with fat mass [48]–[50]. However, despite exceptions (most notably athletes), BMI is closely correlated with the percentage of body fat. Additionally, because it is the most convenient measure available, it is the one most often used both in large-scale studies and in clinical practice [51]–[53]. The findings of the current study can therefore be compared with those of other studies, whether or not their data are limited to Swiss sources. Fourth, occupation is a limited measure of socioeconomic position, particularly for young individuals [54], [55]. A significant percentage of the conscripts had not yet completed their schooling, so they had yet to establish themselves in the labour market. Moreover, the socioeconomic position of parents [56], social networks [57] and neighbourhood [58], [59] can contribute to a young person's socioeconomic background. We aimed at indirectly capturing these factors by adjusting models with tertiles of postcode-level Swiss-SEP index, which serves as an approximate gauge of the socioeconomic status of the area concerned. It should be noted, for instance, that in a recent study of mortality based on the Swiss National Cohort, Swiss-SEP was shown to perform well when used to adjust individual-level SEP. Fifth, postcode of residence was the only available geographical variable in the dataset. In Switzerland, postcodes do not correspond exactly to administrative boundaries, and they change over time. Thus regional variation could not be analyzed unless the postcodes were standardized over time and linked to administrative boundaries. For those cases in which overlap was lacking, we used population weights in order to assign conscripts to the most likely region of residence, which may have turned out to be a neighbouring community of residence instead of the actual one. However, since Swiss postcode geography has a relatively high spatial resolution (N = 3187 on 31 January 2013), the possibility that the postcode alters the membership of a NUTS 2 region on account of the temporal aggregation, a split or a population-weighting assignment is negligible.

The development and current levels of OWOB among recent cohorts of Swiss conscripts add to the picture presented by cross-sectional studies. For instance, Rühli et al. [33] analyzed cantonal variation in average BMI, Saely et al. [60] the association between BMI and metabolic parameters of the voluntary blood sample and Staub et al. [34] the right skewness of the BMI distribution. The current study extended those findings by using a larger sample and providing a more extensive as well as more detailed spatio-temporal description of the trends in median BMI.

The mean BMI of the conscripts born in 1992 (23.4 kg/m^2^) was similar to the value (23.2 kg/m^2^) obtained from the small sample of 165 young men (15–29 years of age) who were measured during the Swiss salt survey in 2011 [18], [19]. In contrast, the BMI of young men (15–24 years of age) who participated in the 2012 SHS was slightly lower (23.6% had a BMI over 25 kg/m^2^, compared with 24.9% of the conscripts born in 1992) [61], possibly because the data were self-reported. It should be noted that the next survey will not be available until 2017 and that the recent participation rate has been steadily declining, from 64% in 2002 to 54% in 2012. Moreover, the signal of the stabilization of the OWOB levels among the most recent cohorts of Swiss conscripts is consistent with the levelling indicated by studies based on schoolchildren data [28], [62].

The results of this study help in the assessment of the trend in Swiss conscripts' BMI at the national level over the long term. **Figure S2** shows mean levels of BMI by conscription year from 1950 to 2003, as published in Staub [63]. We extended these by calculating 2004–2012 yearly means using data on 19-year-old conscripts. While the mean BMI remained relatively stable from the 1950s to the mid-1980s, since then it has climbed, at first steadily but then, during the first decade of this century, sharply. The fact that the BMI increases since then have weakened may a sign that after half a century of increase, the BMI of Swiss conscripts has reached a plateau. Further monitoring and studies will be needed, of course, to determine whether this is the case.

The current study also confirms findings of recent Swiss and international studies indicating that BMI is relatively low among individuals of relatively high socioeconomic status [8], [11], [13]. Moreover, the relatively low BMI values in the western, French-speaking part of Switzerland have already been signalled by the 2012 SHS data [61]. As for the average BMI level of the Swiss conscripts in 2010 (23.4 kg/m^2^), it matches the levels of conscripts in other European countries where analyses based on compulsory conscription data are available (Norway: 23.4 kg/m^2^; Germany: 23.7 kg/m^2^; Austria: 23.4 kg/m^2^) [64]–[66]. The levels of OB (BMI≥30 kg/m^2^) were slightly lower in Switzerland than in the listed countries (5.8% vs. 8.1–8.5%). In Finland, the plateauing of the OWOB increase among conscripts appeared four years earlier (in 2005/2006) than when it was signalled in Switzerland [67], whereas the BMI values of Austrian conscripts showed no signs of stabilization between 2006 and 2010 [65]. However, because conscription procedures and age-at-conscription rules vary from country to country, in the absence of a comparative international study these figures should be treated with caution.

### Conclusions

Switzerland, with its culturally and economically diverse population, provides an ideal opportunity to examine variations in BMI levels over time and to understand the forces driving these variations. The current study shows not only that the rising BMI levels among Swiss conscripts may have begun to stabilize but also that there is a significant temporal and regional variation. What is needed next is an analysis of conscript data at a higher level of geographical resolution. While the data provide little more than clues to the socioeconomic status of the conscripts, they can be linked to other datasets, such as the ch-x Swiss Federal Survey on Lifestyle, Consumption and Future Aspiration [54]. Such linkages offer the potential of time series that are not only longer but also enriched by an increased range of contextual variables.

## Supporting Information

Figure S1 Mean BMI of Swiss conscripts across year of birth and professional status (first panel from top), tertiles of median Swiss-SEP index of postcode of residence (second panel), degree of urbanicity of community of residence (third panel) and region of residence (fourth panel).
(TIFF)

Figure S2 Mean BMI of 19-year-old Swiss conscripts across conscription years 1950–2012. Data for the period 1950–2003 come from Staub (2010); data for the 2004–2012 period come from the conscript records of 19-year-olds in the current study population.
(TIFF)

Table S1 Distribution of the Swiss conscripts across year of birth and age at conscription compared with the population count of 17-year-old Swiss residents in a given year and the total number of live births in a given year. Figures in bold refer to the birth years corresponding to the ones used in this study.
(DOCX)

## References

[1] James PT, Leach R, Kalamara E, Shayeghi M (2001) The worldwide obesity epidemic. Obes Res 9 Suppl 4 228S–233S 10.1038/oby.2001.123 11707546

[2] World Health Organization (WHO) (2013) Obesity and overweight. Fact sheet No 311. Available: http://www.who.int/mediacentre/factsheets/fs311/en/. Accessed 31 January 2014.

[3] Kelly T, Yang W, Chen CS, Reynolds K, He J (2008) Global burden of obesity in 2005 and projections to 2030. Int J Obes 32: 1431–1437 10.1038/ijo.2008.102 18607383

[4] Davin C, Vollenweider P, Waeber G, Paccaud F, Marques-Vidal P (2012) Cardiovascular risk factors attributable to obesity and overweight in Switzerland. Nutr Metab Cardiovasc Dis 22: 952–958 10.1016/j.numecd.2011.01.004 21478001

[5] Engeland A, Bjørge T, Selmer RM, Tverdal A (2003) Height and body mass index in relation to total mortality. Epidemiology 14: 293–299 10.1097/01.EDE.0000047889.30616.73 12859029

[6] Faeh D, Matzke A (2012) Ernährung und Gesundheit. In: Federal Office of Public Health, editor. Sechster Schweizerischer Ernährungsbericht. Bern: Merkur Druck AG.pp. 128–208.

[7] Faeh D (2013) Epidemiologie von Übergewicht und Adipositas bei Zürcher Erwachsenen. Gesundheit, Gesundheitsförderung und Gesundheitswes im Kant Zürich: 1–7.

[8] Galobardes B, Costanza MC, Bernstein MS, Delhumeau C, Morabia A (2003) Trends in risk factors for lifestyle-related diseases by socioeconomic position in Geneva, Switzerland, 1993–2000: health inequalities persist. Am J Public Heal 93: 1302–1309 10.2105/AJPH.93.8.1302 PMC1447961 12893619

[9] Morabia A, Constanza M (2005) The Obesity Epidemic as Harbinger of a Metabolic Disorder Epidemic: Trends in Overweight, Hypercholesterolemia and Diabetes Treatment in Geneva, Switzerland, 1993–2003. Am J Public Heal 95: 632–635 10.2105/2004.047877 PMC1449232 15798121

[10] Faeh D, Bopp M (2010) Increase in the prevalence of obesity in Switzerland 1982–2007: birth cohort analysis puts recent slowdown into perspective. Obesity 18: 644–646 10.1038/oby.2009.310 19779475

[11] Marques-Vidal P, Bovet P, Paccaud F, Chiolero A (2010) Changes of overweight and obesity in the adult Swiss population according to educational level, from 1992 to 2007. BMC Public Health 10: 87 10.1186/1471-2458-1087 20170554 PMC2831837

[12] Tschumper A, Nägele C, Alsaker FD (2006) Gender, type of education, family background and overweight in adolescents. Int J Pediatr Obes 1: 153–160. 17899633 10.1080/17477160600881767

[13] Faeh D, Braun J, Bopp M (2010) Prevalence of obesity in Switzerland 1992–2007: the impact of education, income and occupational class. Obes Rev. doi:OBR793 [pii]10.1111/j.1467-789X.2010.00793.x. 10.1111/j.1467-789X.2010.00793.x 20673278

[14] Wolff H, Delhumeau C, Beer-Borst S, Golay A, Costanza MC, et al (2006) Converging prevalences of obesity across educational groups in Switzerland. Obesity 14: 2080–2088 10.1038/oby.2006.243 17135626

[15] Schneider H, Venetz W, Gallani-Berado C (2009) Overweight and obesity in Switzerland: Cost burden of adult obesity in 2007. Basel: Federal Office of Public Health.

[16] Schutz Y, Woringer V (2002) Obesity in Switzerland: a critical assessment of prevalence in children and adults. Int J Obes Relat Metab Disord 26 Suppl 2 S3–S11 10.1038/sj.ijo.0802122 12174323

[17] Stamm HP, Fischer A, Wiegand D, Lamprecht M (2011) Indikatorensammlung zum Monitoring-System Ernährung und Bewegung (MOSEB) Im Rahmen des Nationalen Programms Ernährung und Bewegung 2008-2012 (NPEB). Indikatorenbericht Stand Oktober 2011. Zürich: Swiss Federal Office of Public Health.

[18] Chappuis A, Bochud M, Glatz N, Vuistiner P, Paccaud F, et al.. (2011) Swiss survey on salt intake: main results. Service de Néphrologie et Institut Universitaire de Médecine Sociale et Préventive (CHUV), editor Lausanne: Federal Office of Public Health.

[19] Ogna A, Forni Ogna V, Bochud M, Paccaud F, Gabutti L, et al.. (2013) Prevalence of obesity and overweight and associated nutritional factors in a population-based Swiss sample: an opportunity to analyze the impact of three different European cultural roots. Eur J Nutr published. doi:10.1007/s00394-013-0643-2. 10.1007/s00394-013-0643-2 24374796

[20] Ledergerber M, Steffen T (2011) [Prevalence of overweight and obesity in children and adolescents from 1977 to 2009 - examination of the school medical data of more than 94,000 school-age children in the city of Basel (Switzerland)]. Gesundheitswesen 73: 46–53 10.1055/s-0030-1268447 21283967

[21] Schneider H, Venetz W, Gallani-Berado C (2009) Overweight and obesity in Switzerland: Overweight and obesity trends in children. Basel: Federal Office of Public Health.

[22] Zimmermann M, Gubeli C, Puntener C, Molinari L (2004) Overweight and Obesity on 6-12-year-old Children in Switzerland. Swiss Med Wkly 134: 523–528. 15517505 10.4414/smw.2004.10640

[23] Finucane MM, Stevens GA, Cowan MJ, Danaei G, Lin JK, et al (2011) National, regional, and global trends in body-mass index since 1980: systematic analysis of health examination surveys and epidemiological studies with 960 country-years and 9.1 million participants. Lancet 377: 557–567 10.1016/S0140-6736(10)62037-5 21295846 PMC4472365

[24] Chiolero A, Peytremann-Bridevaux I, Paccaud F (2007) Associations between obesity and health conditions may be overestimated if self-reported body mass index is used. Obes Rev 8: 373–374 10.1111/j.1467789X.2007.00375.x 17578386

[25] Faeh D, Braun J, Bopp M (2009) Underestimation of obesity prevalence in Switzerland: comparison of two methods for correction of self-report. Swiss Med Wkly 139: 752–756 doi:smw-12863 [pii] smw-12863 19950041 10.4414/smw.2009.12863

[26] Faeh D, Marques-Vidal P, Chiolero A, Bopp M (2008) Obesity in Switzerland: do estimates depend on how body mass index has been assessed? Swiss Med Wkly 138: 204–210 doi:smw-12065 [pii] 2008/13/smw-12065. 18389393 10.4414/smw.2008.12065

[27] Aeberli I, Henschen I, Molinari L, Zimmermann MB (2010) Stabilization of the prevalence of childhood obesity in Switzerland. Swiss Med Wkly 140: w13046 doi:smw-12982 [pii]smw-12982. 20349364 10.4414/smw.2010.13046

[28] Murer SB, Saarsalu S, Zimmermann MB, Aeberli I (2013) Pediatric adiposity stabilized in Switzerland between 1999 and 2012. Eur J Nutr. doi:10.1007/s00394-013-0590-y. 10.1007/s00394-013-0590-y 24121393

[29] Olds T, Maher C, Zumin S, Peneau S, Lioret S, et al (2011) Evidence that the prevalence of childhood overweight is plateauing: data from nine countries. Int J Pediatr Obes 6: 342–360 10.3109/17477166.2011.605895. 21838570

[30] Schneider H, Dietrich ES, Venetz WP (2010) Trends and stabilization up to 2022 in overweight and obesity in Switzerland, comparison to France, UK, US and Australia. Int J Env Res Public Heal 7: 460–472 10.3390/ijerph7020460 PMC2872281 20616985

[31] Stronski-Huwiler S, Stamm HP, Frey D, Christen L, Christen S, et al.. (2013) Übergewicht und Adipositas bei Kindern und Jugendlichen im Kanton Zürich. Gesundheit, Gesundheitsförderung und Gesundheitswes im Kant Zürich: 9–16.

[32] Staub K, Woitek U, Rühli F (2013) Impact and pitfalls of conscription data. In: Hermanussen M, editor. Auxology. Schweizerbart. pp. 146–149.

[33] Ruhli F, Henneberg M, Woitek U (2008) Variability of height, weight, and body mass index in a Swiss armed forces 2005 census. Am J Phys Anthr 137: 457–468 10.1002/ajpa.20889 18668685

[34] Staub K, Ruhli FJ, Woitek U, Pfister C (2010) BMI distribution/social stratification in Swiss conscripts from 1875 to present. Eur J Clin Nutr 64: 335–340 10.1038/ejcn.2010.7. 20160753

[35] Rami B, Schober E, Kirchengast S, Waldhor T, Sefranek R (2004) Prevalence of overweight and obesity in male adolescents in Austria between 1985 and 2000. A population based study. J Pediatr Endocrinol Metab 17: 67–72. 14960023 10.1515/jpem.2004.17.1.67

[36] Toschke AM, Ludde R, Eisele R, von Kries R (2005) The obesity epidemic in young men is not confined to low social classes—a time series of 18-year-old German men at medical examination for military service with different educational attainment. Int J Obes 29: 875–877 10.1038/sj.ijo.0802989. 15917848

[37] Engeland A, Bjorge T, Selmer RM, Tverdal A (2003) Height and body mass index in relation to total mortality. Epidemiology 14: 293–299. 12859029

[38] Engeland A, Bjorge T, Sogaard AJ, Tverdal A (2003) Body mass index in adolescence in relation to total mortality: 32-year follow-up of 227,000 Norwegian boys and girls. Am J Epidemiol 157: 517–523. 12631541 10.1093/aje/kwf219

[39] Logistikbasis der Armee LBA San (2014) Militärärztlicher Dienst. Available: http://www.lba.admin.ch/internet/lba/de/home/themen/sanit/milit.html. Accessed 7 February 2014.

[40] Rühli F, Henneberg M, Woitek U (2008) Variability of Height, Weight and Body Mass Index in a Swiss Armed Forces 2005 Census. Am J Phys Anthr 137: 457–468. 10.1002/ajpa.20889 18668685

[41] Swiss Federal Statistical Office (2014) Bevölkerungsstand und -struktur – Indikatoren. Available: http://www.bfs.admin.ch/bfs/portal/de/index/infothek/onlinedb/stattab.html. Accessed 31 January 2014.

[42] World Health Organization (WHO) (2014) BMI classification. Available: http://apps.who.int/bmi/index.jsp?introPage=intro_3.html. Accessed 31 January 2014.

[43] International Labour Organization (2014) International Standard Classification of Occupations (ISCO). Available: http://www.ilo.org/public/english/bureau/stat/isco/. Accessed 31 January 2014.

[44] Panczak R, Galobardes B, Voorpostel M, Spoerri A, Zwahlen M, et al (2012) A Swiss neighbourhood index of socioeconomic position: development and association with mortality. J Epidemiol Community Health 66: 1129–1136 10.1136/jech-2011-200699 22717282 PMC5204371

[45] Swiss Federal Statistical Office (2014) Nomenklaturen – Räumliche Gliederungen. Available: http://www.bfs.admin.ch/bfs/portal/de/index/infothek/nomenklaturen/blank/blank/raum_glied/01.html. Accessed 31 January 2014.

[46] Koenker R (2005) Quantile Regression: Econometrics, statistics and mathematical economics. Cambridge: Cambridge University Press.

[47] Bottai M, Frongillo EA, Sui X, O'Neill JR, McKeown RE, et al.. (2013) Use of quantile regression to investigate the longitudinal association between physical activity and body mass index. Obesity: 1–8. doi:10.1002/oby.20618. 10.1002/oby.20618 PMC3954962 24039223

[48] Henneberg M, Veitch D (2005) Is Obesity as mesaured by Body Mass Index and Waist Circumference in Adult Australian Women 2002 just a Result of Lifestyle? Hum Ecol 13: 85–89.

[49] Burkhauser RV, Cawley J (2008) Beyond BMI: the value of more accurate measures of fatness and obesity in social science research. J Heal Econ 27: 519–529 10.1016/j.jhealeco.2007.05.005 18166236

[50] Schneider HJ, Friedrich N, Klotsche J, Pieper L, Nauck M, et al (2010) The predictive value of different measures of obesity for incident cardiovascular events and mortality. J Clin Endocrinol Metab 95: 1777–1785 10.1210/jc.2009-1584 20130075

[51] Keys A, Fidanza F, Karvonen MJ, Kimura N, Taylor HL (1972) Indices of relative weight and obesity. J Chronic Dis 25: 329–343. 4650929 10.1016/0021-9681(72)90027-6

[52] Malatesta D (2013) Gültigkeit und Relevanz des Body-Mass-Index (BMI) als Massgrösse für Übergewicht und Gesundheitszustand auf individueller und epidemiologischer Ebene.

[53] Sebo P, Beer-Borst S, Haller DM, Bovier PA (2008) Reliability of doctors' anthropometric measurements to detect obesity. Prev Med 47: 389–393 10.1016/j.ypmed.2008.06.012 18619998

[54] Abel T, Hofmann K, Schori D (2013) Social and regional variations in health status and health behaviours among Swiss young adults. Swiss Med Wkly 143: w13901 10.4414/smw.2013.13901. 24363126

[55] Fuchs VR (2004) Reflections on the socio-economic correlates of health. J Health Econ 23: 653–661 10.1016/j.jhealeco.2004.04.004. 15587692

[56] Smith GD, Hart C, Blane D, Gillis C, Hawthorne V (1997) Lifetime socioeconomic position and mortality: prospective observational study. BMJ 314: 547–547 10.1136/bmj.314.7080.547. 9055712 PMC2126019

[57] Leahey TM, Gokee LaRose J, Fava JL, Wing RR (2011) Social influences are associated with BMI and weight loss intentions in young adults. Obesity 19: 1157–1162 10.1038/oby.2010.301. 21164501 PMC3079776

[58] Oliver LN, Hayes MV (2008) Effects of neighbourhood income on reported body mass index: an eight year longitudinal study of Canadian children. BMC Public Health 8: 16 10.1186/1471-2458-8-16. 18194577 PMC2291462

[59] Kakinami L, Séguin L, Lambert M, Gauvin L, Nikiema B, et al (2014) Poverty's latent effect on adiposity during childhood: evidence from a Quebec birth cohort. J Epidemiol Community Health 68: 239–245 10.1136/jech-2012-201881. 24272921

[60] Saely CH, Risch L, Frey F, Lupi GA, Leuppi JD, et al (2009) Body mass index, blood pressure, and serum cholesterol in young Swiss men: an analysis on 56784 army conscripts. Swiss Med Wkly 139: 518–524. 19795526 10.4414/smw.2009.12653

[61] Swiss Federal Statistical Office (2013) Schweizerische Gesundheitsbefragung 2012 - Übersicht. Neuchatel.

[62] Stamm H, Lamprecht M, Gebert A, Wiegand D (2013) Vergleichendes Monitoring der Gewichtsdaten von Kindern und Jugendlichen in der Schweiz. Analyse von Daten aus den Kantonen Basel-Stadt, Basel-Landschaft, Bern, Genf, Graubünden, Jura, Luzern, Obwalden und St. Gallen sowie den Städten Bern und Zürich.

[63] Staub K (2010) Der biologische Lebensstandard in der Schweiz seit 1800. Historisch-anthropometrische Untersuchung der Körperhöhe (und des Körpergewichts in der Schweiz seit 1800, differenziert nach Geschlecht, sozioökonomischem und regionalem Hintergrund (PhD Thesis, University of Bern).

[64] Staub K, Woitek U, Rühli FJ (2013) Grenzüberschreitende Zusammenarbeit mit anthropometrischen und medizinischen Daten der Rekrutierung. Swiss Rev Mil Disaster Med 1: 41–45.

[65] Poglitsch M, Kefurt R, Mittlböck M, Bohdjalian A, Langer FX, et al (2011) Prevalence of obesity and overweight in male 18-year-olds in Austria from 2006 to 2010: an update. Eur Surg 43: 181–186 10.1007/s10353-011-0009-z

[66] Statistics Norway (2013) Statistical Yearbook of Norway 2013. Oslo.

[67] Bingham CML, Lahti-Koski M, Puukka P, Kinnunen M, Jallinoja P, et al (2012) Effects of a healthy food supply intervention in a military setting: positive changes in cereal, fat and sugar containing foods. Int J Behav Nutr Phys Act 9: 91 10.1186/1479-5868-9-91 22849620 PMC3511183

