# Supplementary figures and images for: Socioeconomic, Temporal and Regional Variation in Body Mass Index among 188,537 Swiss Male Conscripts Born between 1986 and 1992
Source: PLoS One. 2014 May 12;9(5):e96721. doi: 10.1371/journal.pone.0096721 (PMC4018351; doi:10.1371/journal.pone.0096721)

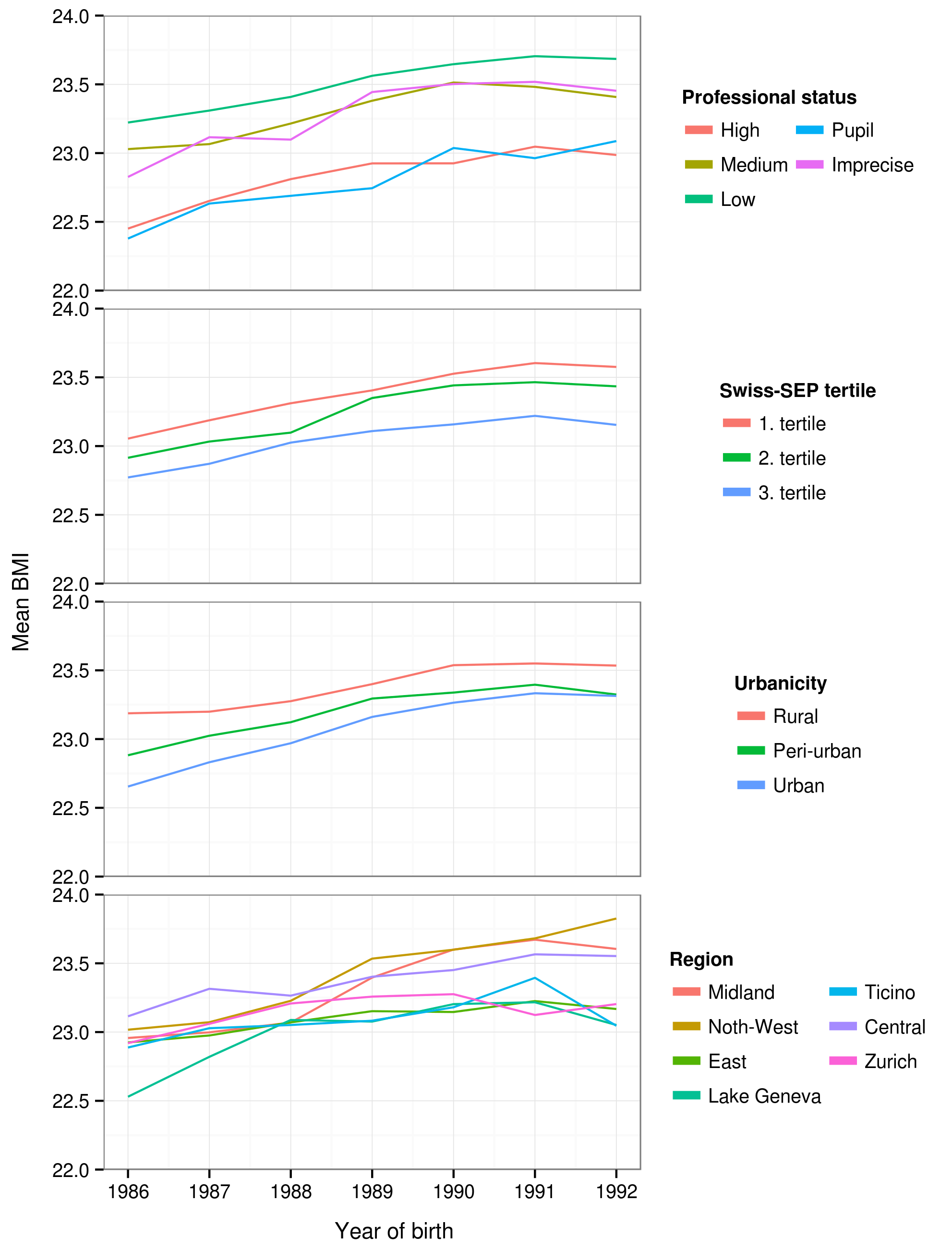

Supplement: Figure S1 — Mean BMI of Swiss conscripts across year of birth and professional status (first panel from top), tertiles of median Swiss-SEP index of postcode of residence (second panel), degree of urbanicity of community of residence (third panel) and region of residence (fourth panel). (TIFF) [file pone.0096721.s001.tiff]

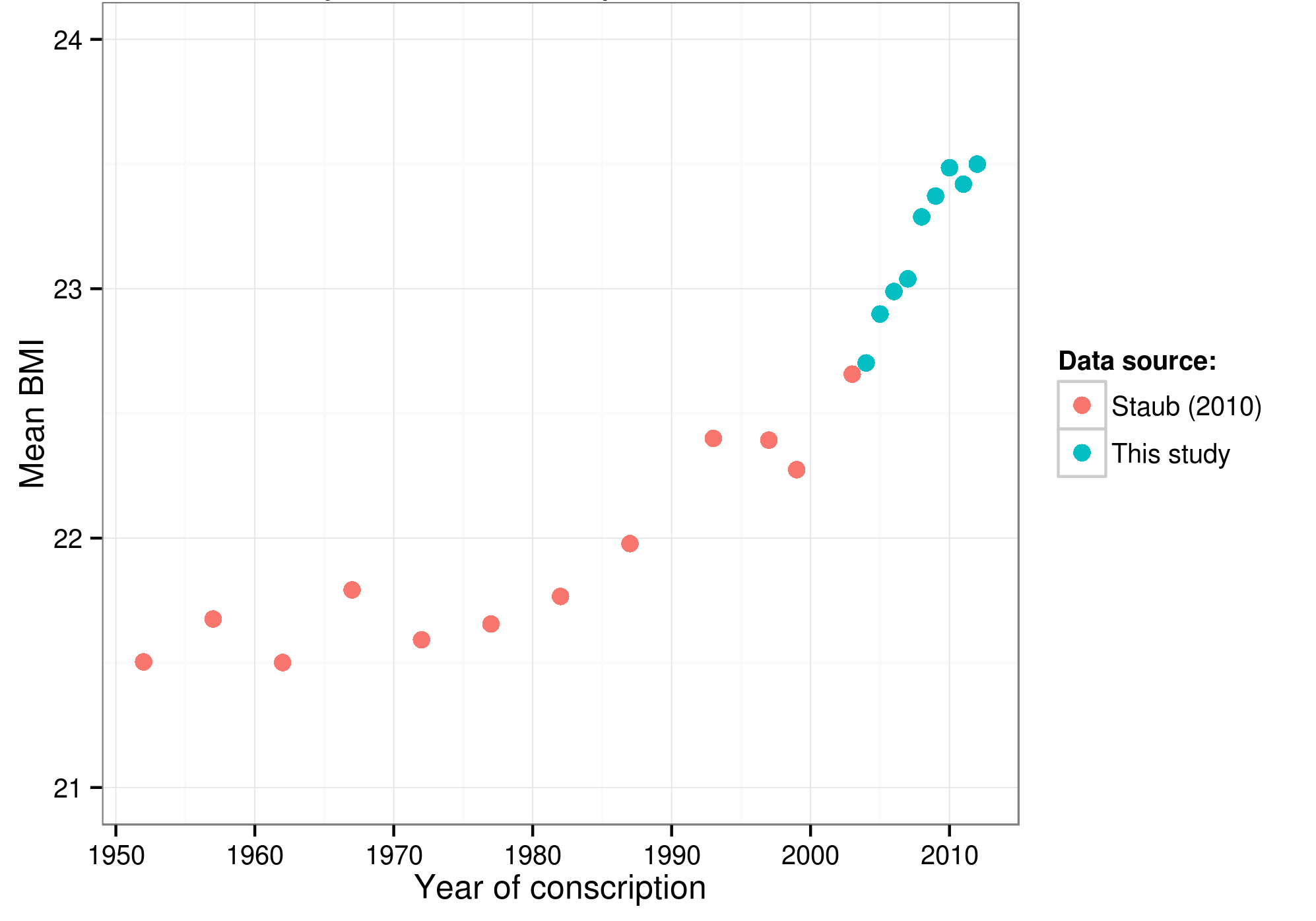

Supplement: Figure S2 — Mean BMI of 19-year-old Swiss conscripts across conscription years 1950–2012. Data for the period 1950–2003 come from Staub (2010); data for the 2004–2012 period come from the conscript records of 19-year-olds in the current study population. (TIFF) [file pone.0096721.s002.tiff]
