# Supplementary material for: Socioeconomic, Temporal and Regional Variation in Body Mass Index among 188,537 Swiss Male Conscripts Born between 1986 and 1992
Source: PLoS One. 2014 May 12;9(5):e96721. doi: 10.1371/journal.pone.0096721 (PMC4018351; doi:10.1371/journal.pone.0096721)
Supplement: Table S1 — Distribution of the Swiss conscripts across year of birth and age at conscription compared with the population count of 17-year-old Swiss residents in a given year and the total number of live births in a given year. Figures in bold refer to the birth years corresponding to the ones used in this study. (DOCX) [file pone.0096721.s003.docx]

| ***Conscription year*** | ***Birth cohort*** | ***Number of conscripts per age group (years)*** | | | | | | ***Population 17 YO*** |  |  | ***Live births*** |  |  |
| --- | --- | --- | --- | --- | --- | --- | --- | --- | --- | --- | --- | --- | --- |
|  |  | ***< 18.5*** | ***[18.5-19.5)*** | ***[19.5-20.5)*** | ***≥20.5*** | ***Total (A)*** | ***18.5-20.5 (B)*** | ***Total (C)*** | ***A / C*** | ***B / C*** | ***Total (D)*** | ***A / D*** | ***B / D*** |
| 2001 | 1984 | 0 | 273 | 5'394 | 6'102 | 11'769 | 5'667 | 34'019 | 34.6% | 16.7% | 32'436 | 36.3% | 17.5% |
| 2002 | 1985 | 306 | 10'832 | 11'818 | 6'723 | 29'679 | 22'650 | 33'887 | 87.6% | 66.8% | 32'023 | 92.7% | 70.7% |
| **2003** | **1986** | **1'691** | **13'166** | **12'350** | **7'435** | **34'642** | **25'516** | **34'723** | **99.8%** | **73.5%** | **32'599** | **106.3%** | **78.3%** |
| **2004** | **1987** | **1'590** | **13'821** | **12'765** | **7'116** | **35'292** | **26'586** | **34'955** | **101.0%** | **76.1%** | **32'787** | **107.6%** | **81.1%** |
| **2005** | **1988** | **2'064** | **14'509** | **12'895** | **7'464** | **36'932** | **27'404** | **36'741** | **100.5%** | **74.6%** | **34'262** | **107.8%** | **80.0%** |
| **2006** | **1989** | **2'371** | **13'749** | **12'940** | **7'744** | **36'804** | **26'689** | **37'000** | **99.5%** | **72.1%** | **34'244** | **107.5%** | **77.9%** |
| **2007** | **1990** | **2'088** | **13'765** | **13'593** | **6'966** | **36'412** | **27'358** | **37'978** | **95.9%** | **72.0%** | **34'491** | **105.6%** | **79.3%** |
| **2008** | **1991** | **2'173** | **14'638** | **13'561** | **4'757** | **35'129** | **28'199** | **38'399** | **91.5%** | **73.4%** | **34'553** | **101.7%** | **81.6%** |
| **2009** | **1992** | **2'504** | **14'452** | **12'348** | **807** | **30'111** | **26'800** | **38'268** | **78.7%** | **70.0%** | **34'005** | **88.5%** | **78.8%** |
| 2010 | 1993 | 2'807 | 12'559 | 2'287 | 0 | 17'653 | 14'846 | 37'061 | 47.6% | 40.1% | 32'547 | 54.2% | 45.6% |
| 2011 | 1994 | 1'792 | 993 | 0 | 0 | 2'785 | 993 | 36'572 | 7.6% | 2.7% | 32'009 | 8.7% | 3.1% |
